# Senescence and Hypoxia Regulate Colon Cancer Cell Transcriptome and Secretome: Insights into Cancer Cell Senescence Pathophysiology

**DOI:** 10.3390/biom16081165

**Published:** 2026-08-11

**Authors:** Chandrasekharam N. Nagineni, Rajani Choudhuri, Murali C. Krishna, James B. Mitchell

**Affiliations:** Molecular Imaging Branch, Center for Cancer Research, National Cancer Institute, Bethesda, MD 20892, USA; chourhur@mail.nih.gov (R.C.); james.mitchell3@nih.gov (J.B.M.)

**Keywords:** senescence, hypoxia, p53, SASP, VEGF, kallikreins, stratifin, cytokines, chemokines

## Abstract

We investigated the effects of senescence and hypoxia on the transcriptome and secretome of the colon cancer cell, HCT-116, in an in vitro model. Senescence was confirmed using SA-β Gal staining and the expression of p53 and p21 proteins, and hypoxia using HIF-1α protein. Control (CN) and senescent (SN) cells were exposed to normoxia or hypoxia, control hypoxia (CH), and senescent hypoxia (SH). Senescence (SN, SH) enhanced the expression of kallikrein-related peptidases, TPp53, p21, optineurin, lipocalin, ADH-1, and stratifin by several folds. Stratifin, with tumor suppressive functions, was upregulated in senescent cells under normoxia but not in hypoxia. Hypoxia (CH and SH) upregulated the expression of many glycolysis genes, especially HK, PFK, aldolase, PDH kinase, and LDH-A. Mitochondrial RNAs (tRNA and rRNA) were increased in SH compared to CH. Significant increases in the secretion of IL-1α, endothelin, bFGF, HB-EGF, PDGF-AB, CCL-5, 7, 22, and CXCL-1 and 8 were observed in SN and SH. VEGF-A, VEGF-C, and TNF-β secretion increased, while PLGF, TGF-α, IL-27, GM-CSF, and M-CSF decreased under hypoxic (CH and SH) conditions. Thus, senescence and hypoxia contribute to cancer cell senescence pathophysiology by regulating the cellular transcriptome and secretome and by both positive and negative feedback mechanisms.

## 1. Introduction

Colorectal cancers (CRCs) are the third most prevalent cancer and the second leading cause of death in the Western world [1,2,3]. The tumor microenvironment (TME) plays critical roles in CRC by regulating oxygen supply, neo-vascularization, metabolic reprogramming, and the activity of immune cells [1,2,4]. The TME is complex and consists not only of cancer cells but also vascular, connective tissue, lymphocytes, macrophages, and senescent cancer cells, as well as extracellular vesicles (EVs) released by these cells [4,5,6,7,8]. The colon microbiota is also associated with CRC, as it can produce inflammatory and toxic molecules which can create a hostile environment [9,10]. Extracellular matrix (ECM) components such as collagen, fibronectin, laminin, and other proteins form a physical scaffold to maintain tumor architecture that controls metastasis processes [4,5,6,8]. The secretion of a variety of cytokines, chemokines, and growth factors by proliferating and senescent cancer cells influences the trafficking of immune cells such as macrophages and lymphocytes, as well as affecting resident cells by both autocrine and paracrine mechanisms [11,12,13,14]. Because of these various factors, the prognosis of CRC depends on the TME, which is dynamic with continuously changing conditions within the tumors [4,8,13,14,15]. A few studies have demonstrated the contribution of the TME, in addition to cancer cell type, oncogenic mutational status, and other factors, as a determining factor in the outcome of CRC prognosis [4,13,14,15].

It is well known that aerobic glycolysis is a characteristic feature of cancer cells; this is commonly known as the Warburg effect [16,17,18]. Due to rapid tumor growth and inefficient vascular reorganization, oxygen supply is limited, resulting in oxygen gradients within the TME [4,5,13,17]. Hypoxia induces activation of HIF-1α that is regulated by prolyl hydroxylase domain-containing enzymes (PHDs) and certain TCA cycle metabolic intermediates that function as oxygen sensors [5,19,20,21]. HIF-1α enhances the expression of genes such as VEGF and other factors for de novo angiogenesis, glycolysis, and other enzymes for metabolic reprogramming [5,19,20,21]. The neo-vasculature is poorly structured and leaky, leading to inefficient oxygen and nutrient delivery to tumor tissue. Hypoxia, caused by elevated glycolysis, causes excess lactic acid to be released into the extracellular space, forming an acidic environment in the TME [19,20,21]. Hypoxia also imposes a barrier to chemotherapy drugs, preventing or limiting their access to the tumors and decreasing cancer cell killing in hypoxic regions. In CRC patients, a positive correlation was observed between HIF-1α and macrophage infiltration in contrast to other solid tumors [13,14,15], indicating a critical role for hypoxia in CRC. Therefore, hypoxic fraction in the TME is another crucial factor that dictates tumor progression or regression by multiple pathways.

Senescence is a permanent loss of proliferative capacity in cancer cells while they retain active metabolic and physiological functions [11,12]. In premalignant and malignant tumor tissue cancer cells, senescence is induced in response to oncogene activation, chemotherapy, radiation therapy, and other DNA-damaging stresses [22,23,24]. Senescent cancer cells have been identified in many premalignant and malignant tumors of different tissues, suggesting their involvement in tumor progression or suppression [25,26]. Characteristic features of senescent cells include elevated expression of p53, p21, p16, and other cell cycle inhibitor genes; enlarged cell size; intracytoplasmic granules; fragmented nuclei; and positive staining for senescence-associated beta galactosidase (SA-β gal) [11,12,27,28]. Another important feature of senescent cells is the secretion of a variety of molecules belonging to cytokines, chemokines, growth factors, and ECM-related proteins, known collectively as senescence-associated secretory phenotype (SASP) [12,27,28,29]. SASP molecules have direct and indirect roles in the maintenance of vascular, extracellular matrix, and immune cell trafficking, as well as cancer cell survival or death and metastasis [12,27,28,29]. Thus, senescence can function as a double-edged sword by inhibiting tumor growth by suppressing the proliferation of potential oncogenic cells, or promoting tumor growth, depending on the origin of tumor tissue and spatial and temporal constraints [30,31,32,33].

CRC tumor tissue is complex, consisting of resident proliferating and senescent cancer cells, vascular and connective tissue cells, and immune cells such as macrophages and lymphocytes [4,13,14,15]. Additionally, pO_2_ gradients at distinct locations within the TME vary, making it more difficult to distinguish specific roles of different cell types [5,13,19,20]. Because of these complexities, it is difficult to delineate the specific roles of proliferating and senescent cancer cell gene expression and patterns of SASP in vivo. Therefore, we used the very well-characterized human colon cancer cell line HCT-116 as an in vitro model to study cellular and molecular biological functions of proliferating and senescent cells under aerobic (20% pO_2_) and hypoxic (1% pO_2_) conditions. Senescence was induced in colon cancer cells by exposing cells to ionizing radiation as described in our recent report [34]. We report here the gene expression and SASP of senescent and proliferating (control) HCT-116 cells exposed to normoxia or hypoxia, and discuss their potential role in tumor pathophysiology and the TME of CRC.

## 2. Material and Methods

### 2.1. Cell Cultures

HCT-116 (ATCC CCL-247, large intestine colon carcinoma), HT-29 (ATCC HTB-38, colon adenocarcinoma), MIA PaCa-2 (ATCC CRL-1420, pancreas carcinoma), and MCF-7(ATCC HTB-22, mammary gland adenocarcinoma) cell lines were obtained from ATCC, Manassas, VA, USA. HCT-116 (ATCC CCL-247) is a human colon carcinoma epithelial cell line derived from the colon of a Caucasian adult male [35]. The HCT-116 cell line expresses wild type genes for TP53, PTEN, and BRAF, but has mutations in codon 13 of the KRAS (G13D) proto-oncogene and PIK3CA (H147R) [36]. HCT-116 cells are positive for carcinoembryonic antigen (CEA) and keratin. HCT-116 cells were grown in Dulbecco’s Modified Eagle’s medium (GIBCO #11995), containing 4500 mg/L glucose, 4 mM L-glutamine, and 1 mM sodium pyruvate. The medium was supplemented with heat-inactivated 10% fetal bovine serum, penicillin (100 u/mL) and streptomycin (100 µg/mL). All cell cultures were maintained at 37 °C in an atmosphere of 5% CO_2_ and 95% air (normoxia). For the hypoxia (1% oxygen) experiments, cells grown under normoxic conditions were transferred to pO_2_ incubator (Panasonic Healthcare, Wood Dale, IL, USA) set at 37 °C, 5% CO_2_, and 1% oxygen by replacing air with nitrogen for the duration of the study.

### 2.2. Protocol for Induction of Senescence by Ionizing Radiation

HCT-116 cells were plated in 60 mm (45–50 k cells) or 100 mm (100 k cells) dishes depending on the experiments. After three days, the medium was replaced with fresh medium and exposed to ionizing radiation (IR) of 2000 cGy (20 Gy) using X-RAD 320 (Precision X-Ray Inc., North Branford, CT, USA). Typically, four days after IR treatment, the media were changed before transferring culture dishes into normoxia or hypoxia incubators, depending on the specific experiment.

### 2.3. Detection of Senescence by Senescence-Associated Beta Galactosidase (SA-β Gal) Staining

Commercially available kits ((BioVision Technologies, Exton, PA, USA, #K320-2500) or Abcam, Waltham, MA, USA, #ab 65351) were used for detecting senescence in cells by SA-β gal staining, according to the procedure recommended by the manufacturer. HCT-116 cells grown in 60 or 100 mm culture dishes were subjected to ionizing radiation, and SA-β gal staining was performed as described in our previous publication [34]. In brief, culture media were removed and washed once with PBS containing calcium and magnesium and incubated with fixative solution for 3–5 min. This was followed by washing twice with PBS, as above, to wash out any adherent fixative. SA-β gal staining solution containing X-GAL (1 mg/mL) dissolved in DMF (dimethyl formamide) was added to fixed cells and incubated at 37 °C in a non-CO_2_ incubator. Blue color staining was observed in senescent cells, and photographs were taken using a KEYENCE microscope (BIOREVO, BZ-9000, Osaka, Japan). Blue-stained and unstained cells were counted typically in ten or more visual fields at separate locations in the same dish, and mean and percentage SA-β gal positive cells were calculated. Typically, more than 95% of irradiated HCT-116 cells were found to be SA-β gal-positive, confirming senescence induction. Representative dishes from the same batch of cultures, control and senescent, under normoxia or hypoxia conditions were used for counting the number of cells using Beckman Coulter Counter, Brea, CA, USA and SA-β gal-positive and enlarged cells were counted by observing them under a microscope.

### 2.4. Cellular Protein Extraction and Quantitation

Cell cultures were washed with ice-cold PBS twice, and cells were scraped into ice-cold PBS. After centrifugation at 2000 rpm for 10 min at 4 °C, cell pellets were then frozen at −70 °C. Cell pellets were incubated with RIPA buffer containing protease and phosphatase inhibitors for 30 min on ice to extract proteins. Samples were centrifuged for 30 min at 15 k rpm and the collected supernatants were used for protein quantitation with the Bio-Rad Laboratories, Hercules, CA, USA, DC protein assay or by Pierce Chemical, Dallas, TX, USA, BCA protein assay.

### 2.5. Immunoblot Analysis of Proteins for p53, p21 and HIF-1alpha

Protein samples were separated on SDS PAGE 4–20% NOVEX gels and transferred to nitrocellulose membranes using the iBlot dry blotting system (Invitrogen, Carlsbad, CA, USA). After blocking in a 2% solution of fat-free dry milk powder, membranes were incubated in relevant primary antibodies followed by washing, and further incubation with appropriate HRP-conjugated secondary antibodies. Membranes were then washed and developed by using chemiluminescence reagents (Thermo Fisher Scientific, Waltham, MA, USA). Protein band images were captured with a Fluor Chem HD2 imager (Alpha Innotech, San Leandro, CA, USA) and processed using image analysis software. The blots were stripped and re-probed for the housekeeping gene proteins actin or HSC 70, to confirm that equal quantities of proteins were loaded into wells. The following primary antibodies were used for Western blots: HIF-1alpha (Abcam, Waltham, MA, USA, #179483), p53 (Santa Cruz Biotechnologies, Dallas, TX, USA, #sc-126), p21 (BD Pharmingen, San Diego, CA, USA, #556431), Actin (Millipore Sigma, Saint Louis, MO, USA #Mab 1501R), HSC 70 (Santa Cruz Biotechnologies, Dallas, TX, USA, #sc-7298).

### 2.6. Collection of Culture Supernatants for Analysis of Secreted Proteins

Since the requirements of growth medium and the patho-physiological characteristics of cancer cells are quite different, serum-free medium was not used for secretome studies to preserve cancer cell function. HCT-116 cells were plated in 60 mm (45–50 k cells) dishes containing complete culture medium with 10% FBS. After three days in culture, the medium was replaced with fresh medium and exposed to ionizing radiation (IR) of 2000 cGy (20 Gy), as described previously [34]. After 4 days in culture, the medium was replaced with fresh medium containing complete culture medium containing 10% FBS. Then, half of the dishes were placed in a hypoxia incubator (37 °C, 5% CO_2_, 1% oxygen), and the others were placed in a standard incubator maintained at 37 °C, 5% CO_2_, and atmospheric air (20% oxygen). After 24 h, culture supernatants were collected, clarified by centrifugation at 2 k rpm for 10 min and frozen at −70 °C until used for analysis (CN, CH, SN and SH sample details are presented in the Supplementary File S1). Corresponding blanks were prepared by keeping fresh culture medium in culture dishes under similar conditions but without cells. The same batch of cell cultures treated under similar conditions were used for cell counts and protein estimation.

### 2.7. Multiplex Luminescence Analysis of Secreted Proteins (Secretome)

Multiplex luminescence assays for protein analytes in culture supernatants were performed by EVE technologies Inc. (Calgary, AB, Canada) using their routine human sample analyte assays. The following panel groups were selected for luminescence assays: Human-Angiogenesis-Growth-Factor-17-Plex, Human-MMP-9-Plex-TIMP-4-Plex, Human-Cytokine-48-Plex, and TGF-beta-3-Plex. All four panels include 81 proteins typically secreted by cells in culture. Quadruplicate samples collected from four independent experiments were used for analysis. Blank values were subtracted from analyte values obtained for control and senescent samples. All results were expressed as pg protein analyte per mg of cellular protein.

### 2.8. Preparation of RNA from Proliferating (Control) and Senescent Cells

HCT-116 cells were plated in 60 mm (45–50 K cells) dishes containing complete culture medium with 10% FBS. After three days in culture, the medium was replaced with fresh medium and exposed to ionizing radiation (IR) of 2000 cGy (20 Gy) as described previously. After 4 days in culture, the media was replaced with fresh medium. Half of the dishes were placed in a hypoxia incubator (37 °C, 5% CO_2_, 1% oxygen) and the other half in a standard incubator (normoxia) maintained at 37 °C, 5% CO_2_, and atmospheric air (20% oxygen). After 24 h, the total RNA was extracted from cells by using RNeasy Mini Kit (QIAGEN, Germantown, MD, USA, #74104) following the manufacturer’s instructions. Control non-irradiated cells similarly exposed to normoxia or hypoxia were used for the preparation of RNA. Total RNAs from control normoxia (CN), control hypoxia (CH), senescence normoxia (SN) and senescence hypoxia (SH) were prepared from four independent experiments. Pooled RNA preparations from these samples were used for RNA-Seq analysis. The quality and quantity of RNA preparations were determined by measuring optical density measurements.

### 2.9. Analysis of Gene Expression and Bioinformatics

NGS sequencing and bioinformatics analysis was performed by LC sciences (Houston, TX, USA) using their propriety technology on their platforms and provided to us. RNA Seq was used for poly A mRNA sequencing after purifying mRNA by using oligo (dt) magnetic beads. Paired-end sequencing was performed on Illumina Novaseq 6000 (Illumina, Inc., San Diego, CA, USA) sequencing system. ***String Tee program*** was used to analyze mRNA expression level by calculating FPKM. The differential expressions of transcripts and genes between two samples were calculated using the R packages ***DESeq*** and ***edgeR***.

### 2.10. Statistical Analysis

Data was analyzed using Microsoft Excel and GraphPad prism software, version 10. *p* values were determined by using Student’s *t* test, and *p* values less than 0.05 were considered as significant. (* *p* < 0.05; ** *p* < 0.01; *** *p* < 0.001; **** *p* < 0.0001).

## 3. Results

In the initial screening studies, we used four different cancer cell lines (HCT-116, HT-29, MIA PaCa-2, and MCF-7) to induce senescence by exposing to ionizing radiation as described in the methods section. After multiple experiments with varying radiation doses and exposure times, the amount of proliferating cancer cells converted to a senescence state was less than 40 percent in HT-29, MIA PaCa-2, and MCF-7 cells, with remaining cells in various states such as proliferation, quiescence, apoptosis or unidentifiable conditions. For HCT-116 cells, conditions were optimized for radiation dose, cell numbers and post-radiation time to achieve more than 90–95 percent cells to senescence as judged by SA-β gal staining (Supplementary File S2). Since our primary objective was to delineate the pathophysiological mechanisms of proliferating and senescent cancer cells, we used the HCT-116 cell line for transcriptome and secretome studies.

### 3.1. Effects of Hypoxia and Senescence on HCT-116 Cells

**Figure 1:**
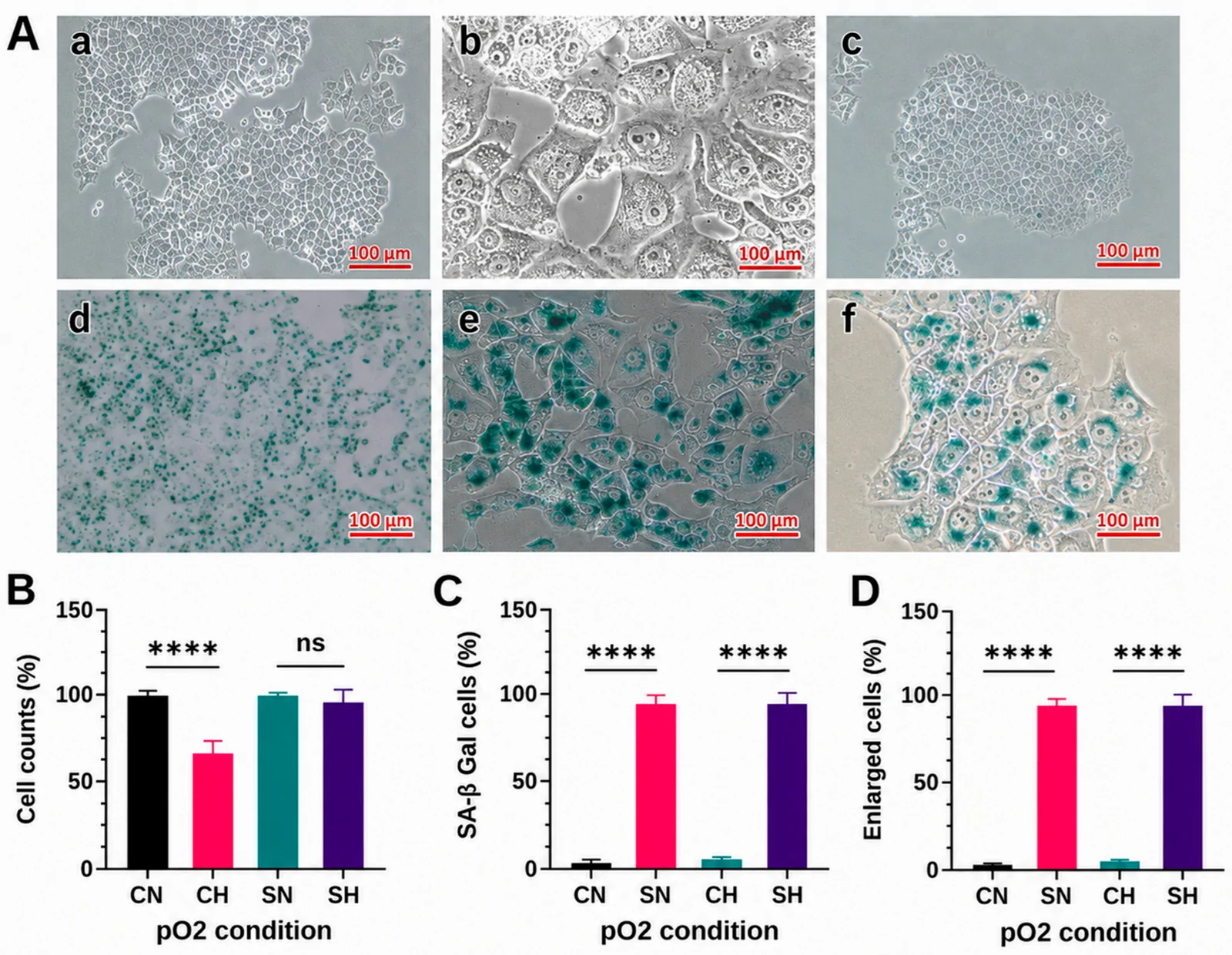
(**A**). Photomicrographs of live control (**a**) and live senescent cells (**b**), SA-β gal-stained control (**c**) and senescent cells at three magnifications (**d**–**f**). (**B**). Characteristics of control (proliferating) and senescent HCT-116 cells under normoxia or hypoxia conditions. Proliferating and senescent cells were exposed to normoxic or hypoxic conditions for 24 h and cell numbers determined by counting using Beckman Coulter Counter. (**C**). Cells fixed and stained for *SA-β gal* positive cells and (**D**). Enlarged cells were counted and shown as percent cells. Results shown are means ± SD of at least 4 independent experiments. Abbreviations used. CN = Control Normoxia; CH = Control Hypoxia; SN = Senescence Normoxia; SH = Senescence Hypoxia. *p* values presented are, ns = not significant, **** < 0.0001. Control (proliferating) and senescence HCT-116 cells at higher magnification are presented in the Supplementary File S3.

We have recently reported the effects of ionizing radiation on senescence in HCT-116 cells [34]. In these studies, conditions were optimized to yield the maximum number of senescent cells to understand the precise pathophysiological mechanisms associated with senescence. The SA-β gal staining method has been used by many investigators to detect senescent cells both in tissue sections and in cultured cells [12,37]. It, along with the expression of p53 and p21 proteins, were used primarily as markers for the presence of senescence in cells. Senescent HCT-116 cells exhibited characteristics of typical senescence, namely enlarged cell size, intracellular granules, and intense positive staining for SA-β gal compared to control cells (Figure 1A). Senescent cells attached to the culture dishes and exhibited viability as evidenced by negative trypan blue staining. Control and senescent cells, after 4 days in normoxia (5% CO_2_ and 20% pO_2_) were transferred to a normoxia or hypoxia (5% CO_2_ and 1% pO_2_) incubator. After 24 h, cells were detached and counted to determine viability and SA-β gal-positivity. Enlarged cells were present in control and senescent cells under normoxia and hypoxia conditions (Figure 1B–D). Under hypoxic conditions, there was a significant decrease in cell numbers in control cells but not in senescent cells (Figure 1B). These results indicate that cell proliferation is inhibited under hypoxic conditions in control cells, since no floating cells were detected. Hypoxia has no effect on senescent cells, as their proliferation abilities are arrested. More than 95% of senescent cells were found to be SA-β gal-positive and enlarged under both normoxia and hypoxia conditions (Figure 1C,D), compared to control cells.

### 3.2. Detection of Senescence and Hypoxia Protein Markers by Immunoblot Analysis

**Figure 2:**
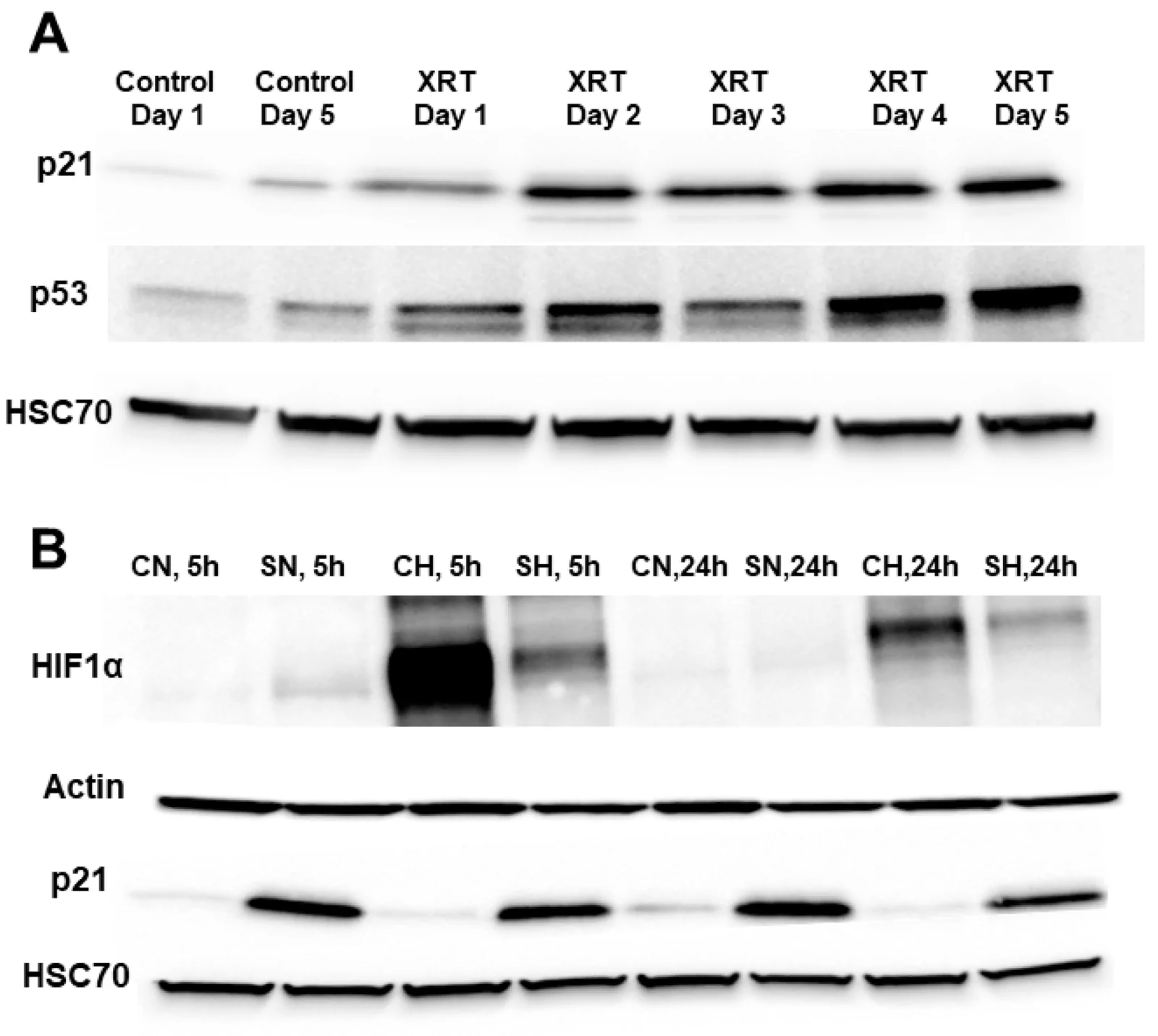
HCT-116 cells were exposed to 20 GY irradiation and cell extracts prepared were used for analysis of various proteins as described in the material and methods section. (**A**). Protein blots for p21 and p53, as senescent cell markers and housekeeping gene HSC70. Both p21 and p53 proteins increased after 20 GY irradiation with maximal intensity at day 4–5. (**B**). Protein blots for HIF1α and housekeeping gene actin in the top 2 lanes and senescent marker p21 and HSC70 in the bottom 2 lanes. Abbreviations, XRT = 20 GY IR; CN = Control Normoxia; CH = Control Hypoxia; SN = Senescence Normoxia; SH = Senescence Hypoxia. Uncropped gel blot pictures are presented in the Supplementary File S4.

HCT-116 cell extracts were prepared from control and irradiated cells after days 1 to 5 to detect senescence makers, p53 and p21 (Figure 2A). Immunoblots show an increased expression of p53 and p21 proteins as a function of time after irradiation, while in control cells they are barely detectable. Similarly, cellular extracts were prepared from control and senescent HCT-116 cells (day 4) exposed to normoxia or hypoxia conditions for 5 or 24 h. Immunoblots for hypoxia marker HIF-1α and senescent cell marker protein p21 are shown in Figure 2B. HIF-1α exhibited strong bands after 5 h under hypoxic conditions, but with a less intense band after 24 h (Figure 2B). Senescent cells exhibited less intense HIF-1α bands compared to control cells both after 5 h and 24 h of exposure to hypoxia. As expected, no positive bands were detected under normoxic conditions, both in control and senescent cells as expected. HSC70 and actin proteins were used as controls.

### 3.3. Gene Expression Results of Control Normoxia (CN) and Hypoxia (CH), and Senescent Normoxia (SN) and Hypoxia (SH)

Box plots and gene expression density plots of CN, CH, SN, and SH are given in the Supplementary File S5. Comparative gene expression profiles of CN, CH, SN, and SH are given in the Supplementary File S6. Volcano plots (Supplementary File S7) describe the levels of expression of genes in HCT-116 cells under various pO_2_ conditions. Comparisons of increase or decrease by more than 2-fold between two treatment conditions are depicted in each Volcano plot. Supplementary File S7A shows the expression of 2-fold up- or downregulated genes between CN and SN. Similarly, CN versus CH, SN versus SH, and CH versus SH are shown in the Supplementary Files S7B, C and D, respectively. There are more upregulated genes compared to downregulated genes under various pO_2_ conditions. The Venn diagram is shown in the Supplementary File S7E depicts the expression of genes under different pO_2_ conditions, showing genes common to all the conditions, as well as unique genes for each treatment condition. There are 19,969 genes expressed in all the conditions, while there were 651 uniquely expressed genes in CN, 832 in CH, 1468 in SN, and 1682 in SH. There were more uniquely expressed genes under hypoxia than normoxia.

**Figure 3:**
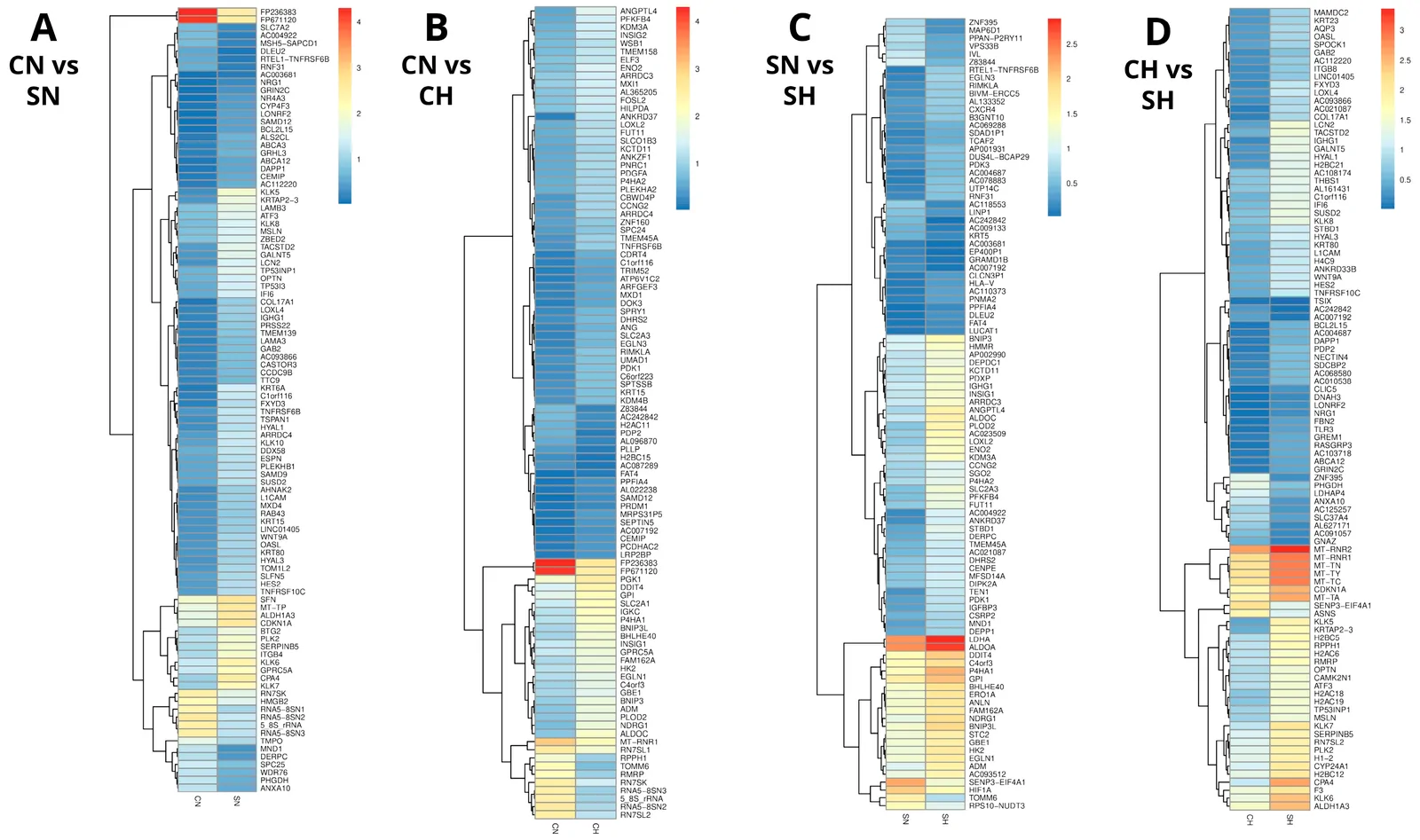
Heat maps related to the expression of the genes in control and senescent HCT-116 cells exposed to the normoxia (20% oxygen) or hypoxia (1% oxygen) conditions. (**A**): CN vs. SN, (**B**): CN vs. CH, (**C**): SN vs. SH, (**D**): CH vs. SH. Abbreviations, CN—control normoxia, CH—control hypoxia, SN—senescence normoxia, SH—senescence hypoxia. Gene expression data related to these heatmaps are presented in the Supplementary Files S8–S11.

Comparative gene expression profiles of CN versus SN, CN versus CH, SN versus SH, and SH versus CH are presented as heat maps in Figure 3. Heat maps identify relative gene expression levels visually based on color intensity. For example, the LNC RNAs FP236383 and FP671120 are highly expressed in proliferating (control) HCT-116 cells (Figure 3A,B right panels) in comparison to control cells in hypoxia and senescent cells under both normoxic and hypoxic conditions. Although these LNC RNAs’ role in cancer is not known, it is interesting to explore. The upregulation of hypoxia-related genes can be clearly seen (Figure 3B,C, left panels) based on color intensity. Senescence-related genes are highly expressed in comparison to control cells (Figure 3A,D, left panels). Gene expression data related to these heat maps are presented in Supplementary Files S8–S11.

### 3.4. Gene Expression Under Normoxia in Control and Senescence HCT-116 Cells

**Figure 4:**
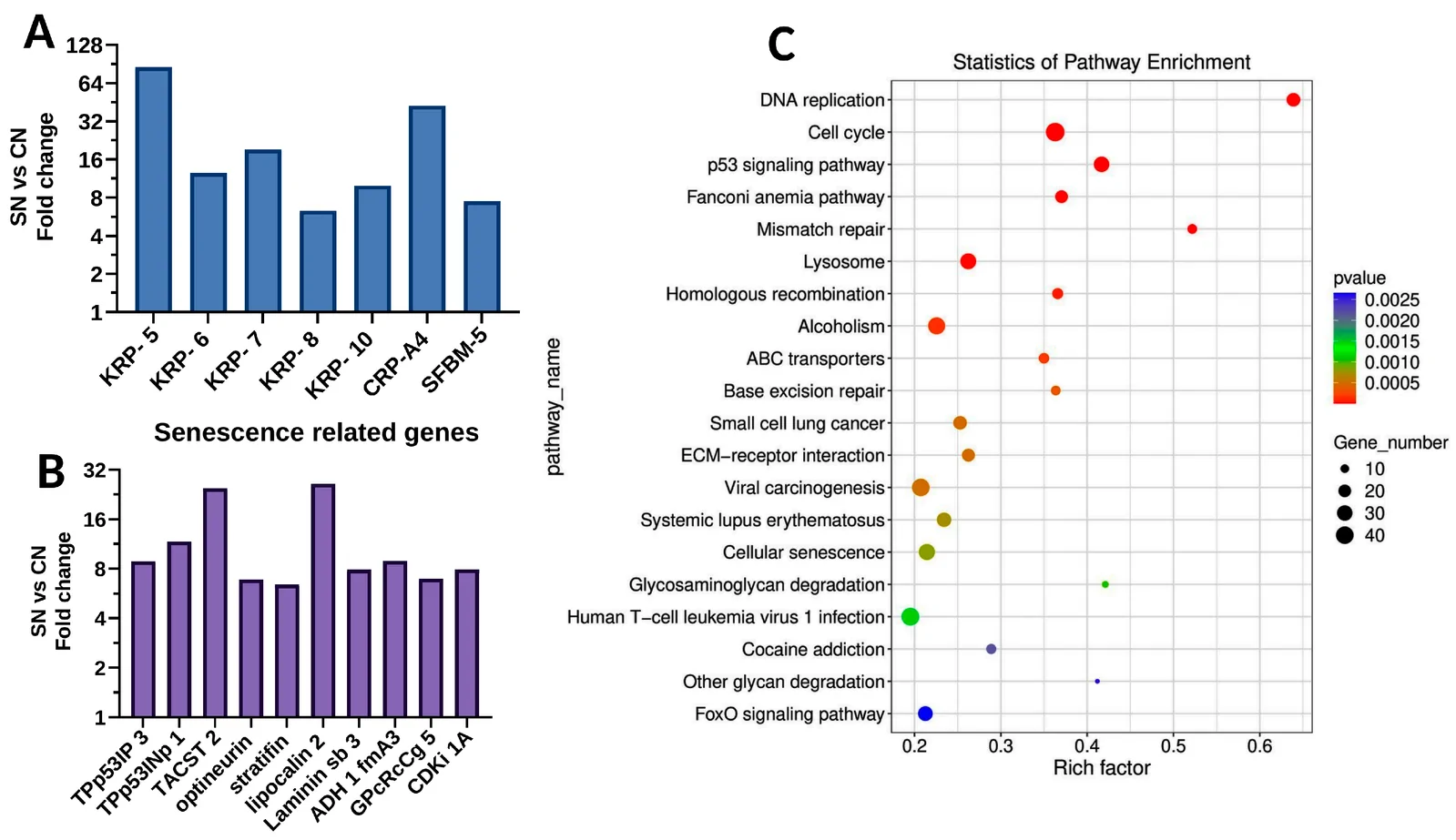
(**A**). Levels of the expression of senescence related protease genes in senescence normoxia (SN) and control normoxia (CN) conditions. Bar graphs are prepared using Graph Pad prism program, version 10, for fold increases with control as 1. Abbreviations, KRP (Kallikerin related peptidases); CRP (Carboxypeptidase); SFBM 5—serpin family B member5. (**B**). Levels of the expression of senescence related genes in senescence normoxia (SN) and control normoxia (CN) conditions. Abbreviations, TPp53 IP3 (tumor protein p53 inducible protein 3); TPp53INp 1(tumor protein p53 inducible nuclear protein 1); TACST2 (tumor associated calcium signal transducer 2); Laminin, sb3 (Laminin subunit beta 3); ADH 1 fmA3 (aldehyde dehydrogenase 1 family member A3); G Pc RCg5 (G protein-coupled receptor class C group 5); CDKi1A (cyclin dependent kinase inhibitor 1A); Not shown in the figure is High mobility group box 2, CN = 185.9, SN = 37.1. (**C**). Pathway enrichment scatter plot comparing senescence normoxia (SN) vs. control normoxia (CN) conditions.

There is a significant upregulation of kallikrein-related peptidases 5, 6, 7, 8, and 10 in senescent cells compared to proliferating control cells (Figure 4A). Carboxypeptidase A4 and serpin family member proteases were also increased by several fold following senescence induction. TPp53-inducible genes, laminin, aldehyde dehydrogenase, stratifin, lipocalin, cyclin-dependent kinase inhibitor and G protein-coupled receptors are upregulated in senescent cells (Figure 4B). In contrast, high mobility group box 2 (HMGB) significantly decreased in senescent cells. The pathway enrichment scatter plot is presented in Figure 4C. DNA replication, cell cycle, p53 signaling, mismatch repair, and lysosome-associated pathways are highly altered during the transition from proliferating phase to senescence stage. Prominent differences are also observed in cellular senescence, ECM-receptor interaction, and Fox O pathways, suggesting their involvement in senescence processes. The comparative gene expression data of CN and SN are presented in the Supplementary File S8.

### 3.5. Effects of Hypoxia on Gene Expression in Control HCT-116 Cells

**Figure 5:**
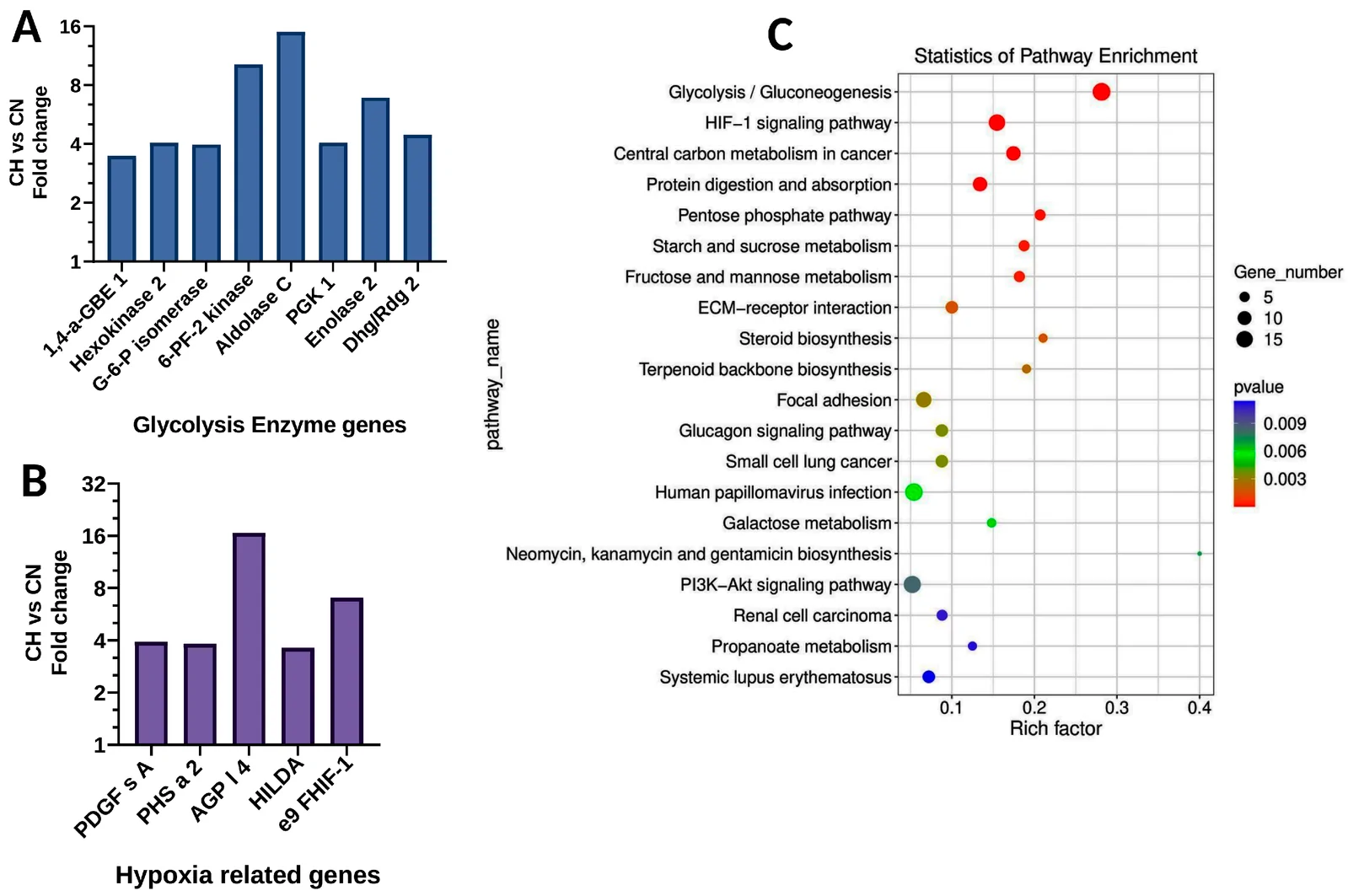
(**A**). Expression of glycolysis related genes in control hypoxia (CH) and control normoxia (CN) conditions. Bar graphs are prepared using Graph Pad prism program for fold increases with control as 1. Abbreviation; 1,4-aGBE 1 (1,4-alpha-glucan branching enzyme 1); G-6-P isomerase (glucose-6-phosphate isomerase); 6-PF-2-kinase (6-phosphofructo-2-kinase/fructose-2,6-biphosphatase); Aldolase C (aldolase, fructose-biphosphate C); PGK-1 (phosphoglycerate kinase 1); Dhg/Rdg 2 (dehydrogenase/reductase 2); Not shown in the figure, translocase of outer mitochondrial membrane 6, CN-168.8, CH-4.8). (**B**). Expression of hypoxia related genes in control hypoxia (CH) and control normoxia (CN) conditions. Abbreviations; PDGF sA (platelet derived growth factor subunit A); PHS a2 (propyl-4-hydroxylase subunit alpha 2); AGP l 4 (angiopoetin like 4); HILDA (hypoxia inducible lipid droplet associated); e9 FHIF-1 (egi-9 family hypoxia inducible factor-1); (**C**). Pathway enrichment scatter plot comparing control hypoxia (CH) vs. control normoxia (CN) conditions.

Proliferating control cells were exposed to normoxia or hypoxia for 24 h, and RNA preparation was subjected to gene expression analysis. Expression of glycolysis genes, hexokinase, phosphofructokinase, aldolase, enolase, and phosphoglycerate kinase increased by several fold in hypoxia cells in comparison to normoxia cells (Figure 5A). In contrast, outer mitochondrial membrane translocase disappeared completely in hypoxia cells. Hypoxia-related genes, propyl-4-hydroxylase, angiopoietin, and other hypoxia-inducible factor-related genes were upregulated in hypoxia cells (Figure 5B). These results are supported by many enhanced pathways, as shown in the pathway enrichment scatter plot (Figure 5C). Pathways enhanced in hypoxia include glycolysis/gluconeogenesis, HIF-1 signaling, central carbon, pentose phosphate, fructose metabolism, and PI3K-AKTsignaling (Figure 5C). The comparative gene expression data of CN and CH are presented in the Supplementary File S9.

### 3.6. Effects of Hypoxia on Gene Expression in Senescent HCT-116 Cells

**Figure 6:**
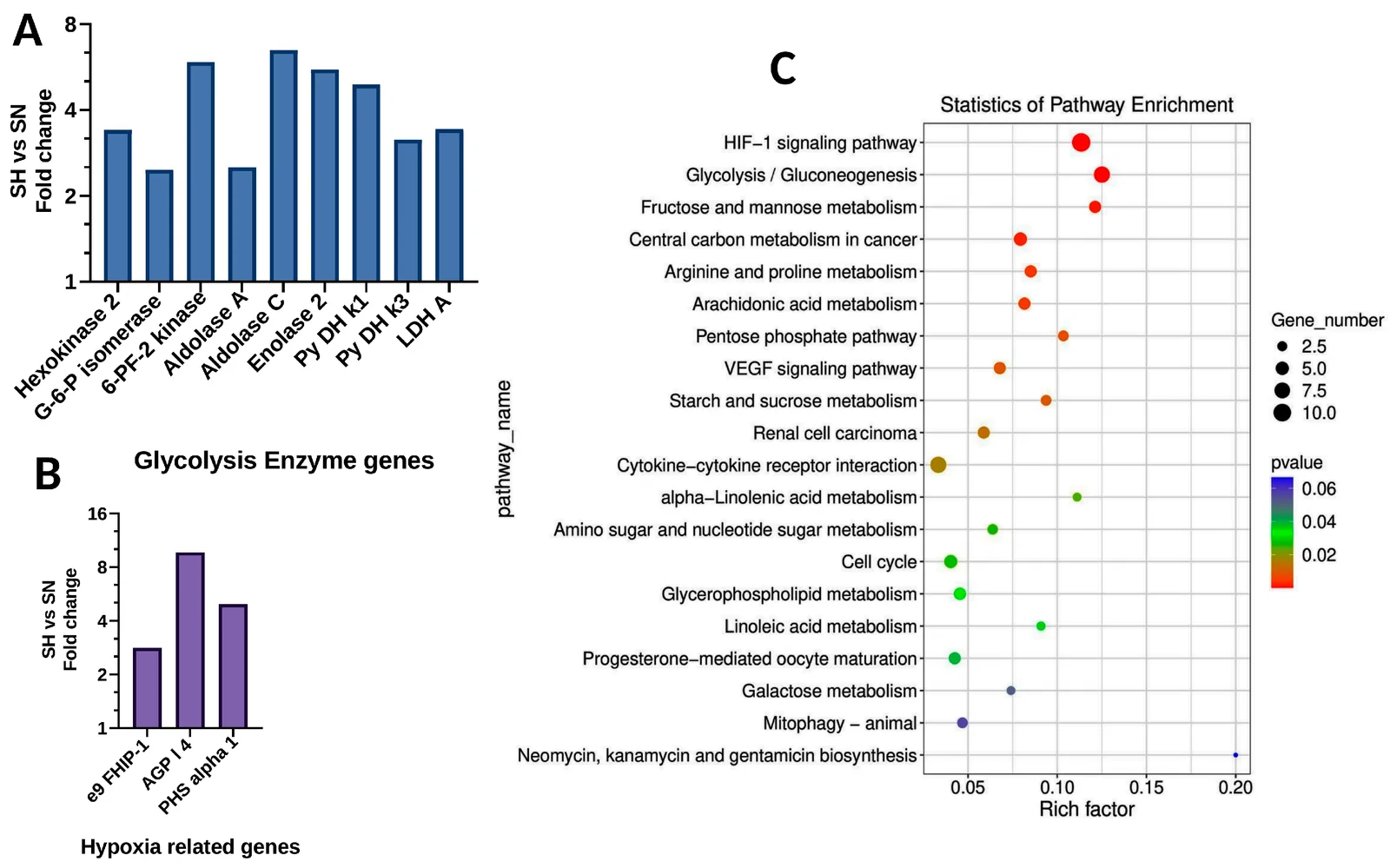
(**A**). Expression of glycolysis related genes in senescence hypoxia (SH) and senescence normoxia (SN) conditions. Bar graphs are prepared using Graph Pad prism program for fold increases with control as 1. Abbreviation; G-6-P isomerase (glucose-6-phosphate isomerase); 6-PF-2-kinase (6-phosphofructo-2-kinase/fructose-2,6-biphosphatase); Aldolase A (aldolase, fructose-biphosphate A); Aldolase C (aldolase, fructose-biphosphate C); Py DH k1 (pyruvate dehydrogenase kinase-1); Py DH k3 (pyruvate dehydrogenase kinase 3); LDH-A (lactate dehydrogenase A). Not shown in the figure: translocase of outer mitochondrial membrane 6, SN = 45.4, SH = 5.0. (**B**). Expression of hypoxia related genes in senescence hypoxia (SN) and senescence normoxia (SN) conditions. Abbreviations; e9 FHIF-1 (egi-9 family hypoxia inducible factor-1); AGP-4 (angiopoietin like 4); PHS alpha-1 (propyl-4-hydroxylase subunit alpha 1). (**C**). Pathway enrichment scatter plot comparing senescence hypoxia (SH) vs. senescence normoxia (SN) conditions.

Senescent cells were exposed to hypoxia or normoxia for 24 h, and RNA samples were subjected to gene expression analysis. Genes upregulated in hypoxic conditions include hexokinase, fructokinase, aldolase, enolase, pyruvate dehydrogenase kinase, and lactate dehydrogenase A (Figure 6A). All these genes are associated with the regulation of glycolysis. Among hypoxia-related genes, angiopoietin, propyl-4-hydroxlase, and ege-9 family HIF-1expression increased (Figure 6B). The pathway enrichment scatter plot (Figure 6C) shows a highly enhanced HIF-1 signaling pathway, glycolysis/gluconeogenesis, fructose, arginine, proline and arachidonic acid metabolism, pentose phosphate, and VEGF signaling pathway. In addition, an increase in cell cycle and cytokine–cytokine receptor interactions are also indicated. The comparative gene expression data of SH and SN are presented in the Supplementary File S10.

### 3.7. Differential Effects of Hypoxia in Senescent and Control HCT-116 Cells

**Figure 7:**
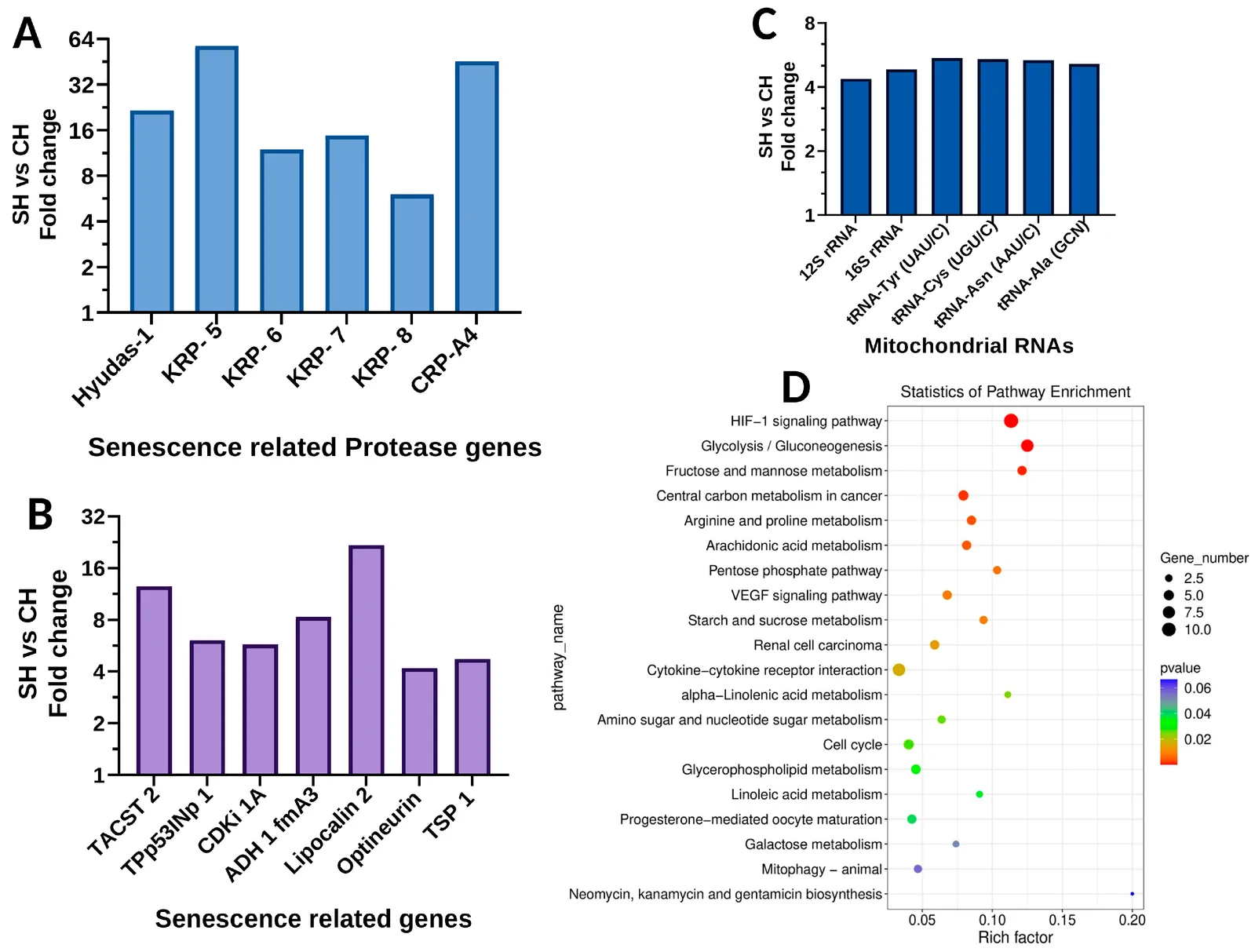
(**A**). Levels of the expression of proteases genes in senescence hypoxia (SH) and control hypoxia (CH) conditions. Bar graphs are prepared using Graph Pad prism program for fold increases with control as 1. Abbreviations, Hyudas-1 (hyaluronidase 1); KRP (Kallikerin related peptidases); CRP (Carboxypeptidase). (**B**). Levels of expression of tumor-related genes in senescence hypoxia (SH) and control hypoxia (CH) conditions. Abbreviations, TACST2 (tumor associated calcium signal transducer 2); TPp53INp 1(tumor protein p53 inducible nuclear protein 1); CDKI 1A (cyclin dependent kinase inhibitor 1A); ADH 1 fmA3 (aldehyde dehydrogenase 1 family member A3); TSP-1 (thrombospondin 1). (**C**). Levels of expression of mitochondrially encoded RNAs in senescence hypoxia (SH) and control hypoxia (CH) conditions. (**D**). Pathway enrichment scatter plot comparing senescence hypoxia (SH) vs. control hypoxia (CH) conditions.

Expression of protease genes was enhanced by several fold in senescent cells under hypoxic conditions, suggesting that hypoxia has minimal effects. Kallikrein peptidase, carboxypeptidase, and hyaluronidase gene expression increased by many folds (Figure 7A). All senescence-related gene expressions also increased under hypoxia to the same extent as under normoxia conditions. As shown in Figure 7B, TPp53-associated genes, cyclin-dependent kinase inhibitor, aldehyde dehydrogenase, lipocalin, and optineurin genes increased by several fold. One important observation made in this study is the four-fold-enhanced expression of mitochondrial ribosomal and tRNAs expression in senescent cells compared to control cells under hypoxic conditions (Figure 7C). The pathway enrichment plot (Figure 7D) demonstrates enhanced HIF-1 and VEGF signaling pathways. Increased metabolic pathways include glycolysis, central carbon and pentose phosphate, arginine, proline, and cytokine–cytokine receptor interaction. The comparative gene expression data of SH and CH are presented in the Supplementary File S11.

### 3.8. Angiogenesis-Related Protein Secretion by Control and Senescent Cells Under pO_2_ Conditions

**Figure 8:**
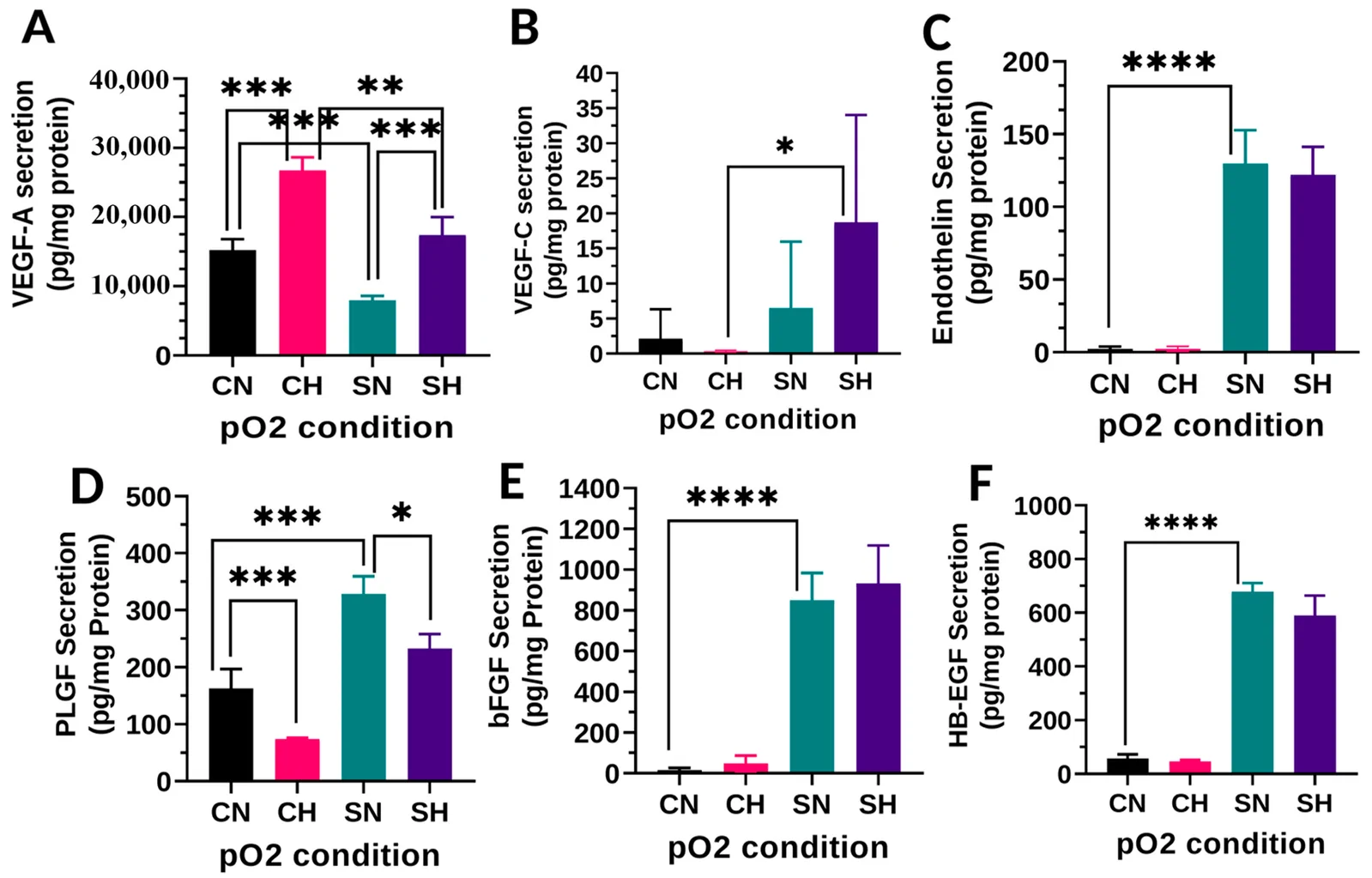
Angiogenesis related proteins secreted by control and senescent HCT-116 cells under normoxia and hypoxia conditions. Culture supernatants were collected after 24 h and the levels of these proteins were determined as described in the methods section. (**A**) Vascular endothelial growth factor-A (VEGF-A); (**B**) Vascular endothelial growth factor-C (VEGF-C); (**C**) Endothelin; (**D**) Placental growth factor (PLGF); (**E**) Basic Fibroblast growth factor (bFGF); (**F**) Heparin-binding EGF-like growth factor (HB-EGF). Abbreviations used. CN = Control Normoxia; CH = Control Hypoxia; SN = Senescence Normoxia; SH = Senescence Hypoxia. Results expressed as pg proteins secreted per mg of cellular protein, are means ± SD of four independent experiments. *p* values presented are, * < 0.05, ** < 0.01, *** < 0.001, **** < 0.0001. Complete lists of quantities of proteins secreted and proteins that were not detectable in all pO_2_ conditions are presented in the Supplementary Files S12 and S13.

VEGF-A is one of the major factors that regulate blood vessel development, growth and functions, thus controlling blood flow and nutrient supply to the tumor. We examined the levels of VEGF-A and other angiogenesis-regulating factors in control and senescent colon cancer cell line HCT-116 cells under normoxic and hypoxic conditions. Among numerous factors secreted, the quantity of VEGF-A secretion is highest in the range of 5000 to 30,000 pg/mg protein (Figure 8A). The secretion of VEGF-A by senescent cells is significantly lower than control cells, and hypoxic conditions significantly elevated the secretion of VEGF-A in both control and senescent cells. VEGF-C secretion was significantly increased under hypoxic conditions by senescent cells (Figure 8B). Endothelin secretion, not detectable in control cells, was enhanced in senescent cells both in normoxic and hypoxic conditions (Figure 8C). PLGF secretion increased in senescent cells compared to control and hypoxia-inhibited secretion of PLGF by both control and senescent cells (Figure 8D). Significant quantities of bFGF were secreted in senescent cells both under normoxia and hypoxia, while this was barely detectable in control cells (Figure 8E). HB-EGF secretion followed along similar lines as bFGF (Figure 8F).

### 3.9. Growth Factor Secretion by Control and Senescent Cells Under pO_2_ Conditions

**Figure 9:**
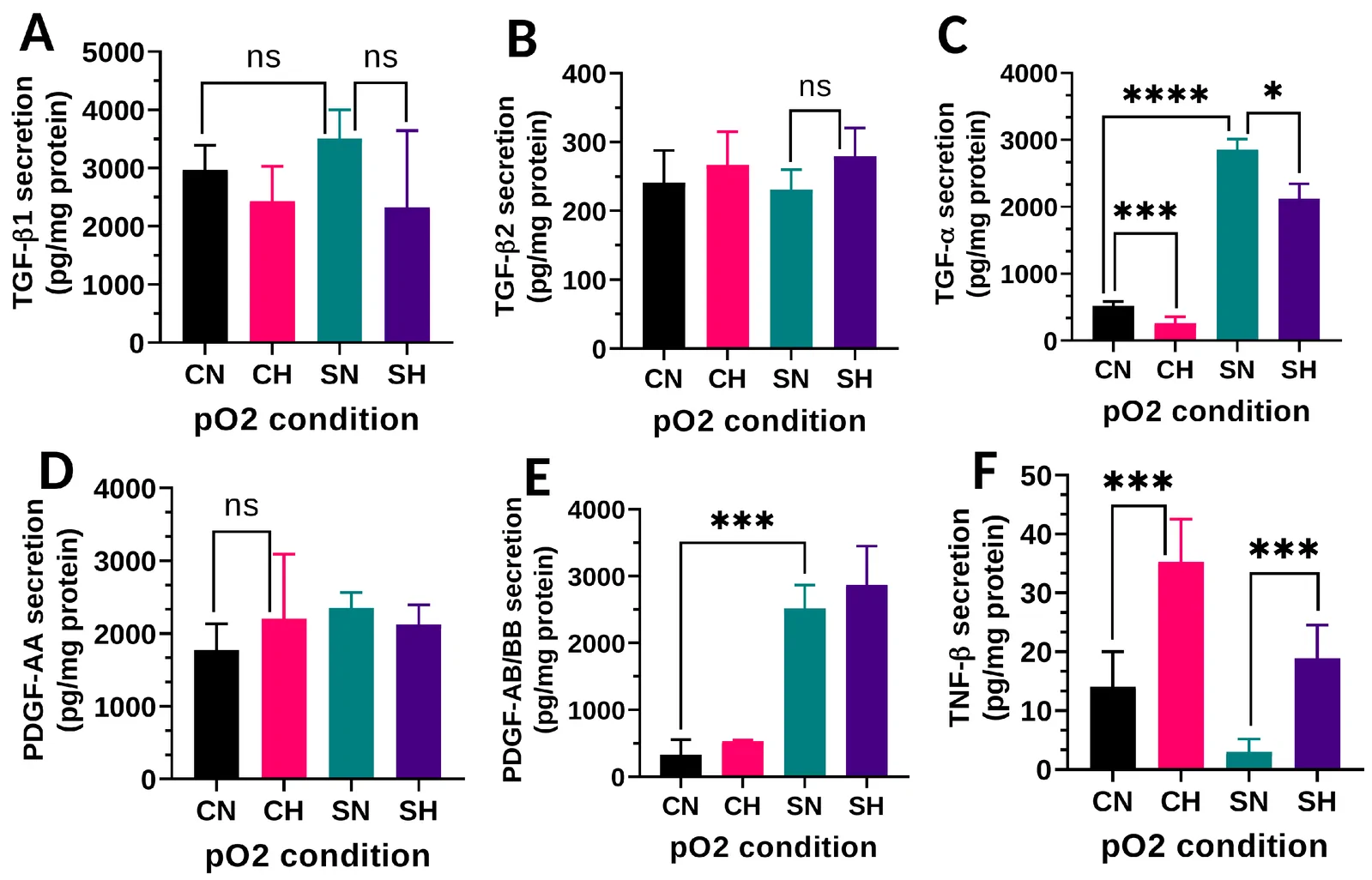
Growth factor proteins secreted by control and senescent HCT-116 cells under normoxia and hypoxia conditions. Culture supernatants were collected after 24 h and the levels of these proteins were determined as described in the methods section. (**A**) Transforming growth factor- beta1(TGF-β1); (**B**) Transforming growth factor-beta2 (TGF-β2); (**C**) Transforming growth factor-alpha (TGF-α); (**D**) Platelet derived growth factor-AA (PDGF-AA); (**E**) Platelet derived growth factor-AB/BB (PDGF-AB/BB); (**F**) Tumor necrosis factor -beta (TNF-β). Abbreviations used. CN = Control Normoxia; CH = Control Hypoxia; SN = Senescence Normoxia; SH = Senescence Hypoxia. Results expressed as pg proteins secreted per mg of cellular protein, are means ± SD of four independent experiments. *p* values presented are, ns = not significant, * < 0.05, *** < 0.001, **** < 0.0001. Complete lists of quantities of proteins secreted and proteins that were not detectable at all pO_2_ conditions are presented in the Supplementary Files S12 and S13.

TGF-β1 secretion was about ten-fold more than the secretion of TGF-β2. Although the secretion of TGF-β2 was slightly decreased under hypoxic conditions, it was not statistically significant (Figure 9A,B). The secretion of TGF-α increased significantly in senescent cells compared to control under normoxic conditions (Figure 9C). Hypoxia slightly decreased the secretion of TGF-α in both control and senescent cells. The secretion of PDGF isoforms exhibits different patterns (Figure 9D,E). PDGF-AA is not different under all four pO_2_ conditions. In contrast, the secretion of PDGF-AB/BB isoforms increased significantly in senescent cells, but hypoxia has no influence on its secretion. Even though small quantities of TNF-β were secreted by control and senescent cells, hypoxia significantly enhanced the secretion of TNF-β in control as well as senescent cells (Figure 9F).

### 3.10. Chemokine Secretion by Control and Senescent Cells Under pO_2_ Conditions

**Figure 10:**
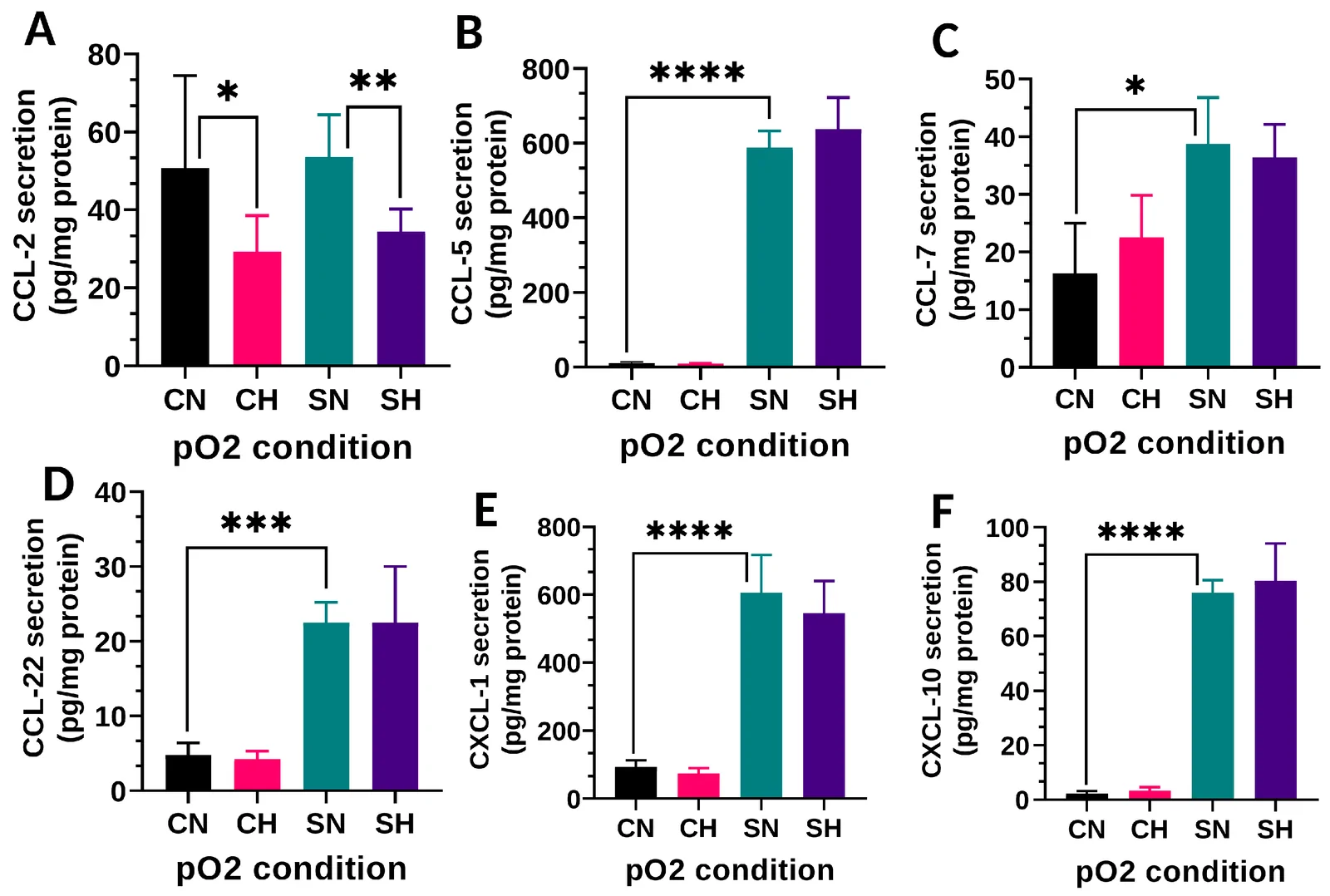
Chemokine proteins secreted by control and senescent HCT-116 cells under normoxia and hypoxia conditions. Culture supernatants were collected after 24 h and the levels of these proteins were determined as described in the methods section. (**A**) CCL-2 (MCP-1); (**B**) CCL-5 (RANTES); (**C**) CCL-7(MCP-3); (**D**) CCL-22 (MDC); (**E**) CXCL-1 (GRO-α); (**F**) CXCL-10 (IP-10). Abbreviations used. CN = Control Normoxia; CH = Control Hypoxia; SN = Senescence Normoxia; SH = Senescence Hypoxia. Results expressed as pg proteins secreted per mg of cellular protein, are means ± SD of four independent experiments. *p* values presented are, ns = not significant, * < 0.05, ** < 0.01, *** < 0.001, **** < 0.0001. Complete lists of quantities of proteins secreted, and proteins that were not detectable at all pO_2_ conditions are presented in the Supplementary Files S12 and S13.

CCL-5 and CXCL-1 secretion are quantitatively different than other chemokines (Figure 10). CCL-2 secretion was not influenced by senescence, but hypoxia significantly decreased in both control and senescent cells (Figure 10A). CCL-5 secretion, not detectable in control cells, was significantly elevated in senescent cells (Figure 10B). Similarly, the secretion of CCL-7 and CCL-22 increased in senescent cells. Hypoxia has no effect on CCL-5, CCL-7, and CCL-22 secretion (Figure 10C,D). Both CXCL-1 and CXCL-10 secretion increased significantly in senescent cells, but hypoxia had no influence (Figure 10E,F).

### 3.11. Interleukin and CSF Secretion by Control and Senescent Cells Under pO_2_ Conditions

**Figure 11:**
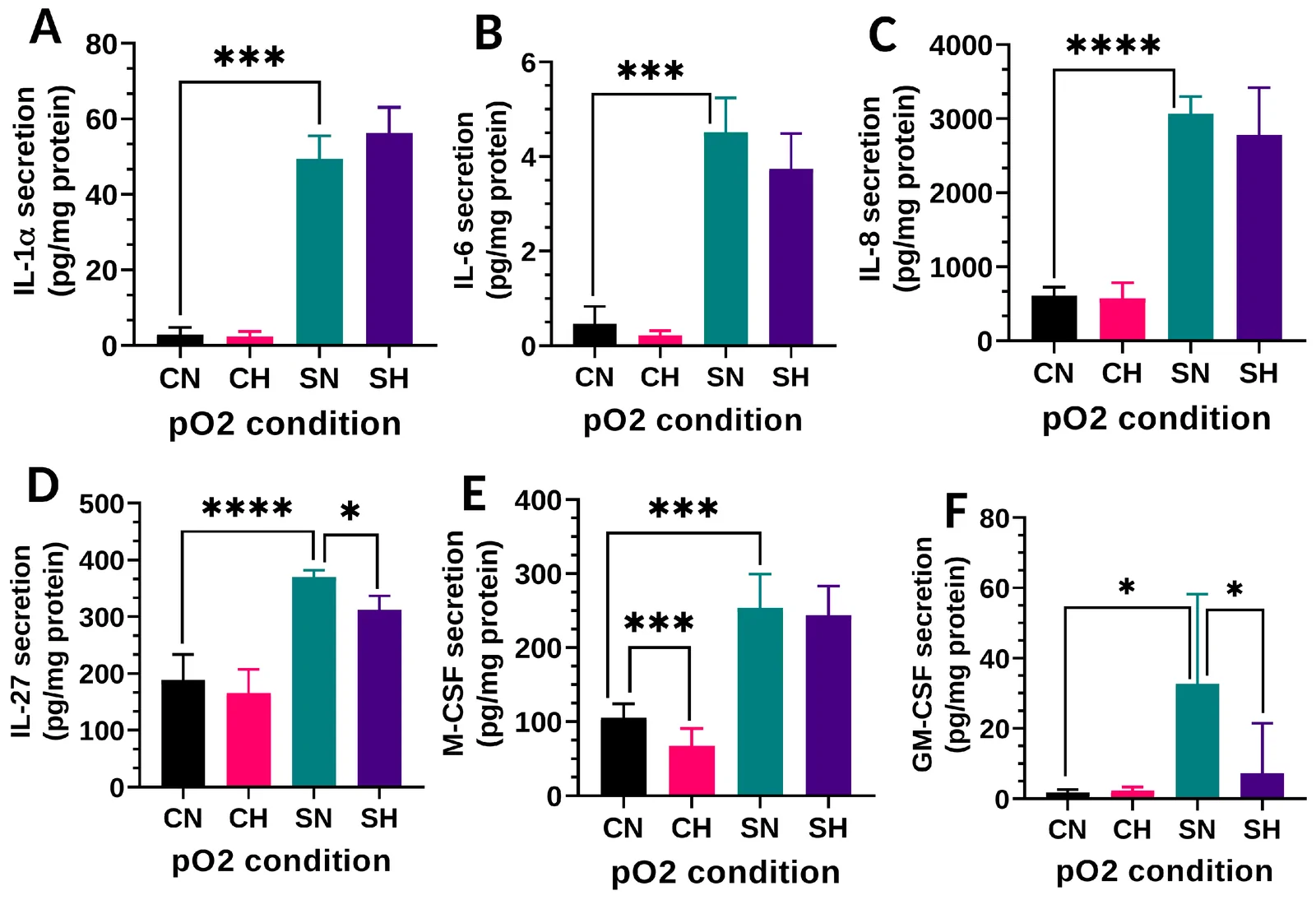
Interleukins and colony stimulating factors secreted by control and senescent HCT-116 cells under normoxia and hypoxia conditions. Culture supernatants were collected after 24 h and the levels of these proteins were determined as described in the methods section. (**A**) Interleukin-1alpha (IL-1α); (**B**) Interleukin-6 (IL-6); (**C**) Interleukin-8 (IL-8); (**D**) Interleukin-27 (IL-27); (**E**) Macrophage colony stimulating factor (M-CSF); (**F**) Granulocyte macrophage colony stimulating factor (GM-CSF). Abbreviations used. CN = Control Normoxia; CH = Control Hypoxia; SN = Senescence Normoxia; SH = Senescence Hypoxia. Results expressed as pg proteins secreted per mg of cellular protein, are means ± SD of four independent experiments. *p* values presented are, * < 0.05, *** < 0.001, **** < 0.0001. Complete lists of quantities of proteins secreted and proteins that were not detectable at all pO_2_ conditions are presented in the Supplementary Files S12 and S13.

Among four interleukins that increased significantly in senescent cells (Figure 11A–D), IL-8 (also known as CXCL-8) secretion was at maximum quantities while lowest secretion was detected for IL-6. IL-1α is the most critical interleukin to initiate and maintain many inflammatory reactions. In senescent cells, IL-1α levels were significantly enhanced, while they were barely detected in control cells (Figure 11A). Significant secretion of IL-27 was detected in senescent cells compared to control cells (Figure 11D). Hypoxia had no significant effect overall on the secretions of all interleukins (Figure 11). Both colony stimulating factors, M-CSF and GM-CSF, secretion significantly increased in senescent cells (Figure 11E,F). Hypoxia effects are seen in M-CSF in control cells, and in IL-27 and GM-CSF in senescent cells.

### 3.12. MMP and TIMP Secretion by Control and Senescent Cells Under pO_2_ Conditions

**Figure 12:**
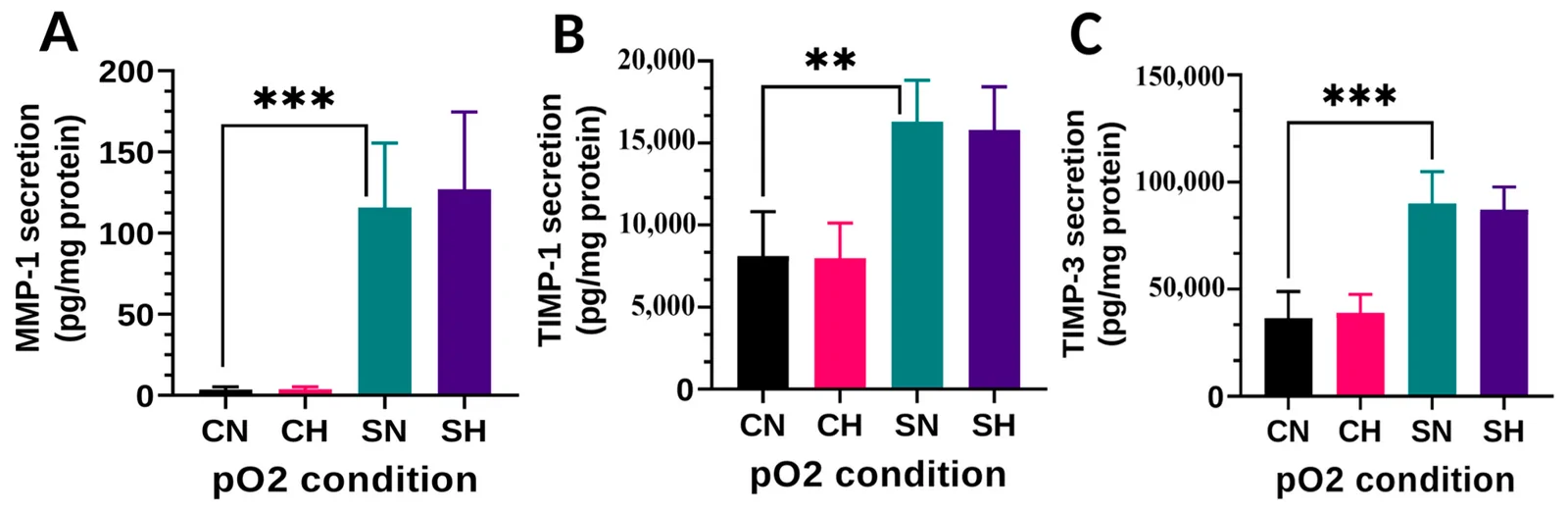
Extracellular matrix (ECM) proteins secreted by control and senescent HCT-116 cells under normoxia and hypoxia conditions. Culture supernatants were collected after 24 h and the levels of these proteins were determined as described in the methods section. (**A**) Matrix metalloprotease-1 (MMP-1); (**B**) Tissue inhibitor of metalloprotease-1(TIMP-1); (**C**) Tissue inhibitor of metalloprotease-3 (TIMP-3). Abbreviations used. CN = Control Normoxia; CH = Control Hypoxia; SN = Senescence Normoxia; SH = Senescence Hypoxia. Results expressed as pg proteins secreted per mg of cellular protein, are means ± SD of four independent experiments. *p* values presented are, ** < 0.01, *** < 0.001. Complete list of quantities of proteins secreted and proteins that were not detectable at all pO_2_ conditions are presented in the Supplementary Files S12 and S13.

Extracellular matrix plays critical roles in the tumor microenvironment in the solid tumors by participating in cancer progression, regression and/or metastasis. Among the ECM-related molecules examined, we found that MMP-1, TIMP-1, and TIMP-3 were significantly increased in senescent cells with no detectable effects under hypoxia (Figure 12).

## 4. Discussion

The tumor microenvironment (TME) plays a critical role in the outcome of cancer suppression or progression and metastasis [5,8,15,21,38]. CRC tumors consist of a heterogeneous collection of proliferating and senescent cancer cells, the vascular system, ECM, secreted factors, and microbial products [4,8,14,20,38]. Senescent cancer cells have been detected in many premalignant and initial stages of primary tumors of prostate, colon adenomas, astrocytoma, and melanocyte nevi, and at various stages of tumorigenesis [25,26,30]. Oncogene-induced senescence in developing tumors may be one of the mechanisms inhibiting uncontrolled cancer cell proliferation and restricting tumor progression [14,24,39]. On the other hand, cells in malignant tumor cells were unable to induce senescence due to lack of active p53 and p16 genes. Senescent cancer cells act by both autonomous and non-autonomous pathways to regulate cell proliferation and or cell cycle arrest through SASP [11,12,27,28]. Hypoxia in the TME creates an acidic environment due to lactic acid accumulation that attracts monocytes for maturation into macrophages and neutrophils [5,19,20,21]. Macrophages and other immune cells in the TME of CRC clears microorganisms and dead and degenerating cells. Extracellular spaces in the TME are filled with several cytokines, chemokines, growth factors, and ECM molecules secreted by tumor-resident and -infiltrating immune cells [15,21,29,32,33]. To understand the colon tumor microenvironment and associated cellular metabolism and physiology, a detailed analysis of colon cancer cell types and tumor tissue samples is required. Transcriptome and secretome analysis is complicated by the complexity of tumor tissue and various cell lines. Therefore, we chose the HCT-116 colon cancer cell line, which was found to be appropriate for cancer cell senescence studies, with more than 90% of proliferating cells reaching senescence following exposure to ionizing radiation (Supplementary Files S2 and S3). In this report, we analyze the role of senescence and hypoxia on the transcriptome and secretome by HCT-116 colon cancer cells and discuss their potential role on cancer cell senescence pathophysiology.

Oxygen gradients within the TME vary depending on the tumor size, vascular bed distribution, fluid perfusion, and other factors [5,17,20,21]. Hypoxic tumors are known to result in poor prognosis due to chemotherapeutic resistance and drug distribution [13,20,21]. Hypoxia within CRC tumors lead to activation of hypoxia-inducible factors (HIF-1) by prolyl hydroxylase domain-containing enzymes (PHDs) and TCA cycle metabolites such as succinate and fumarate, which function as oxygen sensors [5,18,19]. HIF-1 alpha induces several pathways to neutralize hypoxia effects and synthesis of glycolytic enzymes to further augment anaerobic glycolysis to meet increasing energy demands [5,19,20,40,41]. In our study, we found enhanced expression of many of the glycolytic enzymes—hexokinase, phosphofructokinase, aldolase, and others—in both control and senescent cells under hypoxic conditions. In senescent cells under hypoxia, aldolase-A and LDH-A enzymes were prominently elevated compared to control cells, further suggesting that senescent cells respond more intensively to hypoxia for survival. Aldolase enzymes play a significant role in glycolysis by converting 6 carbon fructose1,6 biphosphate into 3 carbon glyceraldehyde-3 phosphate and dihydroxy acetone phosphate molecules [18]. LDH-A is associated with conversion of pyruvate to lactate and conversion of NADH to NAD, while LDH-B converts lactate to pyruvate for the regeneration of NADH from NAD [18]. We have shown in our previous studies that production of lactate by senescent HCT-116 cells is much higher than that of pyruvate [34]. The elevated expression of HK, aldolase-C, GPI, and HIF-1α genes by control HCT-116 cells after 24 h of hypoxia observed in our study is like those reported previously [41].

It is interesting to note that HIF-1 alpha protein expression levels are attenuated at 24 h compared to 5 h both in control rapidly proliferating cancer cells and senescent cells (Figure 2B). The expression of HIF-1 alpha in senescent cells is much lower compared to proliferating cancer cells at 24 h and 5 h time points. Although the precise mechanisms for this discrepancy are not clear from our studies, we can only suggest that cell cycle arrested senescent cancer cells are less responsive to hypoxia in comparison to rapidly dividing cancer cells. It could also be due to metabolic exhaustion during hypoxic conditions in senescent cells. Several transfection factors such as the PHD/EGLN family of oxygen sensors and/or other feedback loops may be responsible for these differential responses for time dependent expression of HIF-1 alpha in control and senescent cells.

The mRNA analysis showed uniquely upregulated expression of genes in control and senescent cells under normoxia or hypoxia. Senescence induced highly elevated expression of p53, p21 genes and proteins, demonstrating their role in stable cell cycle arrest in the G2 phase, which is essential for senescence. p53 is known as guardian of the genome due to its significant role in maintaining genome integrity and preventing tumorigenesis by regulating the cell cycle [11,12,27,39,42]. In senescent cells, cell cycle, p53, mismatch repair and other DNA associated pathways are active, as demonstrated by the gene enrichment plots of HCT-116 cells in our study. Other molecules significantly upregulated in senescence include optineurin, lipocalin 2, and aldehyde dehydrogenase-1 A3 (ADH-1A3). The loss of optineurin was reported in the initial stages of CRC and hepatocellular carcinoma [43]. Optineurin deficiency leads to impaired MHC-1 expression and T cell immunity, thereby affecting immunotherapy efficiency. The major actions of optineurin include autophagy, mitophagy with lysosomal degradation pathway for removing damaged cellular organelles, and unfolded protein aggregates [43,44]. It is interesting to note that, in many cancer cell lines, optineurin levels are low compared to non-cancerous cells [43]. ADH-1A3, belonging to the ADH family of 19 functional genes, is an enzyme that detoxifies exogeneous and endogenous aldehydes into carboxylic acids through NAD or NADP [45,46]. Many aldehydes present in the gut are potentially toxic to humans and microbiota, and require detoxification by ADHs. ADH-1A3 is expressed in HCT-116, HT-29, and caco2 colon cancer cells, and is known to be involved in multiple metabolic pathways like glycolysis, fatty acid, and amino group amino acids [45,46]. Elevated levels of optineurin and ADH-1A3 in senescent HCT-116 cells in our study suggest their role in cancer suppressive actions of senescent cells. Lipocalin-2 (LCN-2, NGAL) is a soluble secretory protein that acts as a negative regulator of cell migration, invasion, and metastasis by controlling EMT and fibrosis processes [47]. Expression of LCN-2 in HCT-116 cells is low but upregulated in senescent cells, suggesting its potential role as a cancer-suppressive factor in CRC.

Two molecules of interest regarding senescence under normoxia and hypoxia are stratifin (SFN) and laminin sb3 (Laminin). Expression of these proteins is upregulated in senescence under normoxic conditions, but not under hypoxic conditions. Laminin family proteins are basement membrane glycoproteins in the ECM associated with type IV collagen networks via entactin and fibronectin [48]. Thus, laminins contribute to cell attachment, differentiation, cell shape, tissue phenotype, and survival. Upregulated expression of laminin in senescent cells indicate their role in stabilizing the phenotype and for proper maintenance of the ECM. SFN (14-3-3 sigma) is a 28–33 kDa acidic polypeptide expressed in stratified epithelial cells known to be used as a coating material for breast implants and vascular stents [49,50]. It is a versatile factor binding more than 100 diverse cellular proteins to regulate the cell cycle, apoptosis, cytoskeleton, and malignant transformations [49,50]. SFN exhibits tumor-suppressive actions and frequently silenced in human epithelial cancers. SFN is downregulated in the breast, stomach, colon, liver, and prostate, and downregulation leads cancer cells to escape from senescence [49,50]. SFN is a negative regulator of cell cycle progression by binding to CDK2 and CDK4 to block cell cycle. It also regulates MDM2 ubiquitination and degradation to activate p53. Induction of SFN in senescent HCT-116 cells suggests the potential role of SFN as another factor for suppression of cancer cell growth in tumors. To our knowledge, this is the first report to suggest SFN as one of the markers of senescence in cancer epithelial cells.

Kallikreins (KLKs) and other peptidases are upregulated in senescence HCT-116 cells. Kallikreins are small protein molecules of 25–30 kDa serine proteases, expressed and secreted by many tumor cells, and circulating molecules that are primarily used as cancer biomarkers [51,52,53]. KLKs 4–8 is connected to ECM degradation, invasion, and metastasis. KLK-6 generates small-molecule antiangiogenic peptides to inhibit angiogenesis. Expressions of KLK 6, 8, and 10 were detected in malignant tumors [52,53]. Overall, KLKs in the TME are associated with ECM degradation, reorganization as well as participation in angiogenesis and metastasis. To our knowledge, there are no reports of elevated expression of KLKs in senescent cancer cells. We observed several-fold increases in the expression of carboxypeptidase A4 (CPA.4) in senescent HCT-116 cells under both normoxia and hypoxia. CPA.4 belongs to the family of exopeptidases, and releases carboxy terminal amino acids from peptides and proteins. CPA.4 is overexpressed in many malignant tumors, including CRC tumors, and overexpression is correlated to poor prognosis [54]. CPA.4 is known to act by histone hyperacetylation, STAT-3, and ERK pathways to promote cancer cells and tumor growth. Increase in CPA.4 in senescent cells may be one mechanism to release free amino acids for augmented metabolic requirements of the cells in the TME, while at the same time promoting growth of proliferating cancer cells.

SASP expressions and secretion in normal tissue cells are extensively studied, but only limited reports are available regarding SASP in cancer cells [12,27,29,34]. SASP comprises many cytokines, chemokines, growth factors, and ECM-related MMPs and TIMPs [12,27,29,34]. SASP secretion is regulated by NFkB and CCAT/enhancer binding protein family (C/EBPs) pathways, by inflammatory initiators and p53, respectively [12,29,42]. These mediators in turn act by regulating many transcription factors, and p38 MAPK, JAK-STAT, and cGAS-Sting pathways. Cytokines and chemokines function as chemoattractants for trafficking immune cells for innate and adaptive defensive mechanisms to perform pro- or antitumor functions [12,15,27,28,33]. In addition, many bio-active lipids act by paracrine and autocrine mechanisms to trigger inflammatory reactions, smooth muscle contraction, and vasodilation, influencing the TME [32,33,55].

VEGF-A and VEGF-C are growth factors for vascular and lymphatic vessel endothelial cells, respectively [20,56]. Among the growth factors associated with angiogenesis, VEGF-A, also known as vascular permeability factor, is secreted by HCT-116 cells in large quantities. Hypoxia significantly increased VEGF-A secretion by both control and senescent cells. However, the secretion of other growth factors, endothelin, bFGF, PDGF-AB, and HB-EGF are increased in senescent cells, but hypoxia had no significant effects. PDGF, bFGF, and EGF are growth factors for smooth muscle, fibroblast, and other cells, and promote the formation and maintenance of new blood vessels and capillaries [56,57]. During hypoxia, angiogenesis processes are active in the TME, promoting neovascularization and thereby restoring oxygen and nutrient supply, as well as anticancer drug delivery to increase the efficiency of chemotherapy [20,56,58]. Contrary to other growth factors, hypoxia decreased PLGF secretion by control and senescent cells. PLGF is overexpressed in CRC cell lines and has been shown to enhance the migration of cells for metastatic invasion [59]. TGF-β1 is secreted in an inactive preform that is activated by proteolysis. Active TGF-β has many roles in the TME, including blood vessel and ECM maintenance as well as epithelial–mesenchymal transitions (EMT), thus contributing to TME architecture [60,61].

Many cytokines and chemokines play pivotal roles in the trafficking and activation of immune cells, macrophages, lymphocytes, neutrophils into the TME for controlling cancer cell promotion or killing [27,28,29,32,33,62]. Quantitatively, IL-8 (CXCL-8) secretion was maximal compared to all other cytokines and chemokines. Levels of IL-8 in cancer cells are higher compared to normal crypt cells in CRC, and higher circulating levels of IL-8 in CRC patients suggest IL-8 as a prognostic marker [63]. CXCL-8 binds to receptors CXCR-1 and CXCR-2 and chemotactically attracts macrophages into the TME for paracrine actions on cancer cells. IL-1α, even in small quantities, acts as a primer to initiate and perpetuate a series of signaling pathways to produce many molecules associated with the maintenance or termination of cellular actions. IL-1 primarily activates NFkB pathways for the expression of many cytokines and chemokines [29,62]. IL-27acts on T cells, NK cells, macrophages, and other immune cells to initiate anti-apoptotic and anti-inflammatory pathways [62,64,65]. However, the anti- or pro-tumor effects of IL-27 are dependent on the type of cancer cells and tumor tissue. Anti-tumor effects of IL-27 have been demonstrated, as evidenced by reduced tumor growth of CRC, prostate, pancreatic, and lung cell mouse xenograft studies [65]. The enhanced expression of IL-27 by senescent HCT-116 cells in our present study supports tumor-suppressive actions of senescence in the TME in CRC.

The mitochondrial genome contains circular DNA with 37 genes encoding 13 proteins belonging to oxidative phosphorylation and 22 tRNAs and 2 rRNA [66]. The expression of mitochondrial transfer and ribosomal RNAs was upregulated in senescent cells under hypoxic conditions compared to control cells. Under hypoxic conditions, senescent cells may activate mitochondrial protein synthesis machinery to enhance oxidative phosphorylation. Hypoxia may also induce reactive oxygen species (ROS) and leads to mitochondrial fission and mitophagy as a protective mechanism to reduce further oxidative injury to the cells.

One novel finding identified in our study is the highly upregulated levels of two novel long non-coding RNAs (LNC RNA), FP236383 and FP671120, in proliferating HCT-116 cells under normoxic conditions but not under hypoxic conditions. These LNC RNAs were not detected in senescent cells under normoxia or hypoxia (Figure 3A,B and Supplementary Files S8–S11). The expression of these LNC RNAs was reported in nasal epithelial cells (airway, transitional) in human SARS-CoV-2 patients [67]. The role and actions of these LNCs are not currently known, but it will be interesting to explore their new and unexpected functions in cancer and other cells during senescence processes.

## 5. Conclusions

The tumor microenvironment (TME) is dynamic and comprises proliferating and senescent resident cells, as well as infiltrating immune cells. In this study, we used an HCT-116 colon cancer cell line as an in vitro model to evaluate the pathophysiology of senescent cells. Senescence was induced by exposing cells to irradiation to obtain more than 90% senescent cells to enable the comparative analysis of proliferating and senescent cells. Transcriptome studies have shown significantly elevated levels of the number of kallikrein-related peptidases in senescent cells with no observable effects of hypoxia. The other major factors significantly increased in senescent cells are stratifin, optineurin, and lipocalin, molecules that have profound effects on carcinogenic processes. Gene enrichment scatter plot analysis indicated significant increases in p53 and cell cycle-dependent pathways in senescence. Hypoxia significantly increased most of the glycolysis enzyme mRNA levels in both proliferating and senescent cells. Under hypoxic conditions, aldolase-A, pyruvate dehydrogenase, and LDH-A enzymes were specifically upregulated in senescent cells, suggesting that glycolysis is effectively regulated during senescence. During senescence, copious quantities of CCL-5 (RANTES), CXCL-1 (GRO-α), and CXCL-8 (IL-8) chemokines are produced that regulate trafficking of immune cells to the TME. The transcriptome and secretome profiles of proliferating and senescent cancer cells under normoxic and hypoxic conditions indicate their potential roles in cancer cell senescence pathophysiology.

## Data Availability

All data related to this article are contained in the main manuscript and Supplementary Files.

## Supplementary Materials

The following supporting information can be downloaded at https://www.mdpi.com/article/10.3390/biom16081165/s1, File S1: Details of experimental samples; File S2: Optimizing conditions for senescence induction by IR in HCT-116 cells; File S3: Photomicrographs of control and senescent cells stained with SA beta Gal staining; File S4: Uncut gel blot images; File S5: Gene expression density and box plots; File S6: Gene expression data of CH, CN, SH and SH samples; File S7: Volcano plots and Venn diagram related to gene expression; File S8: Gene expression, fold changes between CN and SN samples; File S9: Gene expression, fold changes between CN and CH samples; File S10: Gene expression, fold changes between SN and SH samples; File S11: Gene expression, fold changes between CH and SH samples; File S12: Proteins secreted by HCT-116 cells under all pO_2_ conditions; File S13; Proteins not detected in all samples under all pO_2_ conditions.

## Abbreviations

CRC	Colorectal cancer
ECM	Extracellular matrix
HCT-116	Human colon cancer cells
TME	Tumor microenvironment
IR	Ionizing radiation
cGy	cGray units, radiation intensity
SA-β Gal	Senescence associated β-galactosidase
TCA	Tricarboxylic acid cycle
PFK	Phosphofructokinase
Aldolase	Fructose 1,6 biphosphate aldolase
VEGF	Vascular endothelial growth factor
SASP	Senescence-associated secretory phenotype
IL	Interleukin
MMPs	Matrix metalloproteases
TIMPs	Tissue inhibitors of MMPs
