# Supplementary material for: Senescence and Hypoxia Regulate Colon Cancer Cell Transcriptome and Secretome: Insights into Cancer Cell Senescence Pathophysiology
Source: Biomolecules. 2026 Aug 11;16(8):1165. doi: 10.3390/biom16081165 (PMC13510958; doi:10.3390/biom16081165)
Supplement: Supplementary file 1 [file biomolecules-16-01165-s001.zip › Nagineni CN et al. Suppl file S13, Proteins not secreted by CN, CH, SN, SH samples.pdf]

Proteins not detected in all the samples under all po_2 conditions

The following proteins were tested and were not detected in the culture supernatants of control (proliferating) and senescent HCT-116 cells incubated under normoxia or hypoxia conditions. Details of the assay conditions and assay methods are described in detail in the material and methods secretion.

1. **TGF-beta panel: TGF-β**
2. **MMPs and TIMPs panel: MMP-2, MMP-3, MMP-7, MMP-8, MMP-9, MMP-10, MMP-12, MMP-13, TIMP-3, TIMP-4**
3. **Angiogenesis Panel: Angiopoetin-2, EGF, Endoglin, FGF-1, Follistatin, G-CSF, HGF, Leptin, VEGF-D, BMP-9**
4. **Cytokine & Chemokine panel: Eotaxin (CCL-11), FLT3L, Fractalkine (CX3CL1), GM-CSF, IFN-α2, IFN-γ, IL-1β, IL-1RA, IL-2, IL-3, IL-4, IL-5, IL-7, IL-9, IL-10, IL-12p40/P70, IL-15, IL-17, IL-18, IL-22, IL-25, MIP-1α, MIP-1β**
